# Metabolite Profiling and Bioactivities of Leaves, Stems, and Flowers of *Rumex usambarensis* (Dammer) Dammer, a Traditional African Medicinal Plant

**DOI:** 10.3390/plants12030482

**Published:** 2023-01-20

**Authors:** Chiara Spaggiari, Laura Righetti, Costanza Spadini, Giannamaria Annunziato, Aimable Nsanzurwimo, Clotilde Silvia Cabassi, Renato Bruni, Gabriele Costantino

**Affiliations:** 1 Department of Food and Drug, University of Parma, 43124 Parma, Italy; 2 Department of Veterinary Science, University of Parma, 43126 Parma, Italy; 3 INES-Ruhengeri, Institute of Applied Sciences, Musanze 00000, Rwanda

**Keywords:** traditional healers knowledge, medicinal plants, *Rumex usambarensis* (Dammer), Dammer, untargeted metabolomics, UHPLC-TWINS-QTOF

## Abstract

The comprehensive identification of secondary metabolites represents a fundamental step for the assessment of bioactivities and pharmacological properties of traditional herbal drugs. *Rumex usambarensis* (Dammer) Dammer has been described as a multipurpose remedy in different African traditional pharmacopoeias, but its phytochemical profile has not been properly investigated. Herein we report a high throughput metabolomic screening, based on ultra-high performance liquid chromatography-travelling wave ion mobility spectrometry quadrupole time-of-flight (UHPLC-TWINS-QTOF), which was performed for the first time on different *R. usambarensis* plant parts. By applying high-resolution mass spectrometry-based metabolomics and chemometric analysis, a complete discrimination of different aerial parts was obtained, with the annotation of 153 significant metabolites in leaves, stems, and flowers, suggesting an easy authentication and discrimination route. Phytochemical data were correlated to antimicrobial and antioxidant properties. Flavonoids, benzopyranes, chromones, and xanthones derivatives, along with a richer phytocomplex, might be responsible for the stronger bioactivities obtained from flowers.

## 1. Introduction

Plants are still a relevant source of traditional health remedies in developing countries, but for many of them ethnobotanical reports have not been complemented with adequate investigations. For instance, a comprehensive identification of secondary metabolites is often lacking, despite being a crucial starting point for planning a rational use of medicinal plants [1,2]. At the same time, a precise profiling of the different herbal drugs obtainable from a single plant species is relevant for standardization purposes, to prevent adulterations and to evaluate potential uses of less valuable or less available plant material [3]. This is particularly relevant when the traditional use relies on raw extracts aimed at exploiting potential synergies between different phytochemicals.

Medicinal plants are usually investigated according to two main, distinct strategies: one, rooted in modern pharmacology, is focused on pinpointing the role of well-defined and carefully dosed compounds, assumed responsible of the majority of observed biological effects [4,5]. The other tries instead to consider the so-called phytocomplex, that is the whole phytochemical content of a raw extract. Such an approach usually refers to a combination of substances whose chemical structures and abundances are not always comprehensively known [6]. While their research route is different, these approaches are closely related, as in both cases the final goal is a rational evaluation of ethnopharmacological uses, the latter being dependent on the accurate knowledge of the actual phytochemical profile of precise plant organs or parts. *Rumex* genus includes approximately 200 herbaceous species distributed worldwide, and at least 28 of them are traditionally used as remedies in different countries and cultures against a variety of ailments [7]. This sometimes implies very different, if not opposite, putative mechanisms (as in the case of constipation and diarrhea) and spans between pain management, skin and liver diseases, inflammation, and hypertension [8,9,10]. Only a quarter of Rumex species have been the object of joint bioactivity and phytochemical investigations, the latter revealing the presence of multiple chemical classes, mostly represented by polyphenols as phenolic acids and flavan-3-ols (both in monomeric form and as A-type or B-type procyanidins), along with less common substances such as xanthones and chromenones, anthraquinones, naphthalene-1,8-diols, and stilbenoids [11,12]. From a chemotaxonomical standpoint these compounds are not ubiquitous within the genus and may be present only in precise taxa, thus motivating the presence of different bioactivities in separate species. This reinforces the need for accurate phytochemical profiling and its pairing with bioactivities; furthermore, their distribution is often uneven within different parts of the same plant, thus urging a precise evaluation of plant organs. *Rumex usambarensis* (Dammer) Dammer (Polygonaceae) is a climbing shrub found in montane grassland, open mist forest, and bushland throughout the highlands of tropical Africa. It is known for its medicinal uses, these being the infusions of its roots used to treat coughs, rheumatism in the management of chronic joint pains, and different stomach illnesses [13]. Aerial parts are squeezed and the juice used as eye drops, as an anthelminthic or antifungal, and to treat tonsillitis [14,15,16,17,18]. In Rwanda, a poultice obtained from aerial parts of different Rumex species, including *R. usambarensis*, are used to treat common skin diseases or dyssentheria [18,19]. Only a portion of the multiple indications provided by ethnopharmacological reports have been scientifically scrutinized and, in most cases, limited attention has been paid to phytochemical content and to the correlation between bioactivities and profiling. Furthermore, no differential evaluations of different plant organs were made [16,20]. As reported by Vasas et al. in their recent and comprehensive review of the ethnopharmacological knowledge of Rumex genus, most bioactivity studies were conducted on poorly characterized extracts, thus affecting both repeatability and further investigations aimed at maximizing the reported effects [7]. As a result, only a few secondary metabolites have been associated with this plant in the past and no in-deep phytochemical analysis has been performed so far, with emodin, physcion, and chrysophanic acid being the sole secondary metabolites mentioned in root in very outdated reports [21]. No exhaustive investigation on the composition of aerial parts of *R. usambarensis* seems to be available at present, hindering a rational evaluation of the plant properties and limiting both repeatability of results and authentication efforts. While in the past the description of the so called phytocomplex and of its contribution was a more challenging task, the emergence of research tools designed to evaluate the complexity of medicinal plants is helping to overcome previous limitations [22,23,24]. For instance, the availability of metabolomic techniques based on mass spectrometry allowed the simultaneous detection of entire molecular groups and their correlation to bioactivities, paving the way for more precise and reliable pharmacological investigations [25]. To fill such a gap and help further investigations, a comprehensive untargeted metabolomics approach was applied to the leaves, flowers, and stems of *Rumex usambarensis* (Dammer) grown in Rwanda. A discrimination of the extracts and the contribution of main chemical classes with antioxidant and antimicrobial properties was performed by chemometric analysis. Moreover, to provide a proper benchmark and compare Rumex with a natural, polyphenolic extract with a known and consolidated antioxidant and antimicrobial activity, we decided to use green tea from *Camellia sinensis* var Yakubita as positive control. Green tea is investigated in depth and commercially used for its healing, antiseptic, and antioxidant activity, as reported by [26,27,28]. We purposedly chose not to use standard antibiotics, as plant extracts traditionally obtained from *Rumex usambarensis* do not properly represent an alternative to these drugs.

## 2. Results and Discussion

### 2.1. Multivariate Modeling

**Figure 1:**
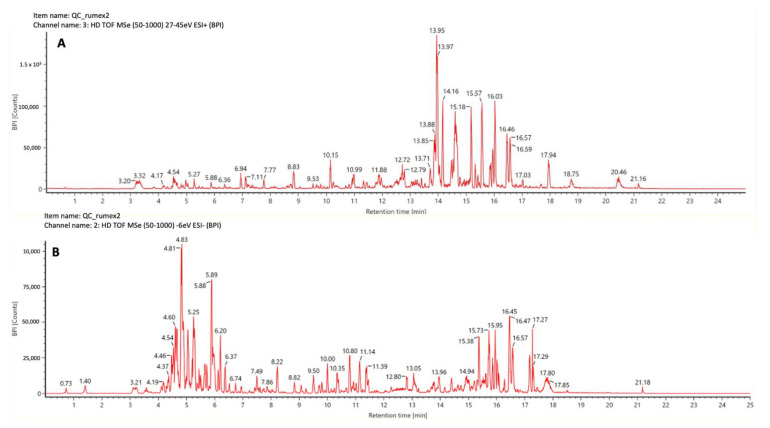
Ultra-high performance liquid chromatography coupled to high resolution tandem mass spectrometry base peak chromatograms of QC pool of *Rumex usambarensis* extract obtained using positive (**A**) and negative (**B**) ionization modes.

An untargeted metabolomics approach based on ultra-high performance liquid chromatography-travelling wave ion mobility spectrometry quadrupole time-of-flight (UHPLC-TWINS-QTOF) was used to explore the metabolome signature of *Rumex usambarensis* leaves, stems, and flowers. Datasets, obtained in positive and negative ionization modes (see Figure 1), were separately submitted for data analysis. A total of 12,677 and 5855 features were initially peak picked for positive and negative modes, respectively.

At this point, the principal components analysis (PCA) models were built to investigate the metabolome, and therefore, the differences between the samples. The mechanism is based on the ability of the PC model to cluster samples in an unsupervised approach, b ecause no information on group identity is used to construct the model. The PCA scores plot obtained is depicted in Figure S1. The three plant organs are nicely grouped in the PCA scatter plot, suggesting metabolome differences within the investigated groups.

Subsequently, significant features were selected, retaining those presenting, simultaneously, fold change > 2 and ANOVA FDR-adjusted *p*-value (q-value) < 0.01. This filtering step returned a dataset with 8355 significant features for positive and 2487 for negative ion mode, which were subjected to the identification. This last step is considered the bottleneck of the whole metabolomics workflow, which remains a major analytical challenge. With exact mass, mass fragmentation, and CCS matching, 153 identifications were assigned belonging to 12 biochemical classes. All significant features are reported, for completeness, in Table S1. Significant metabolites with their statistical values are reported in Table S2. This result also suggests that an easy identification of botanicals and preparations obtained from distinct aerial parts of *R. usambarensis* may be obtained and used for authentication purposes when needed.

**Figure 2:**
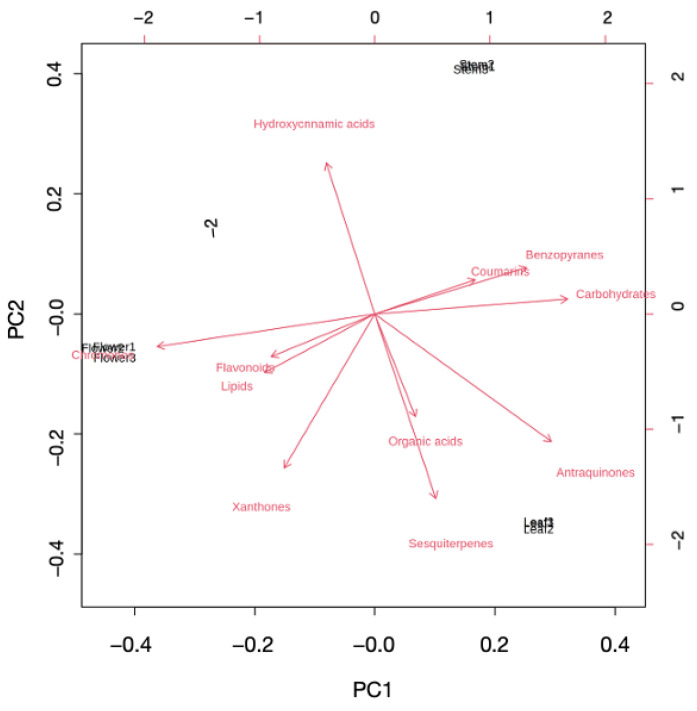
Biplot built using the intensities of annotated metabolites (PC1 54.3%, PC2 38.6%) grouped into the main biochemical classes.

At this point, another PCA model was built using the 153 significant metabolites that were annotated in the study (see Figure 2).

### 2.2. Investigating Metabolome Diversity of Stems, Leaves, and Flowers

**Figure 3:**
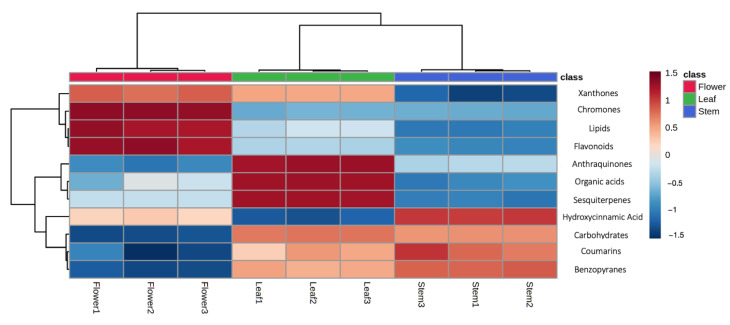
Hierarchical clustering analysis and heat map visualization obtained considering the three plant organs and using the metabolites annotated grouped in biochemical classes (distance: Euclidean; clustering algorithm: Ward).

**Figure 4:**
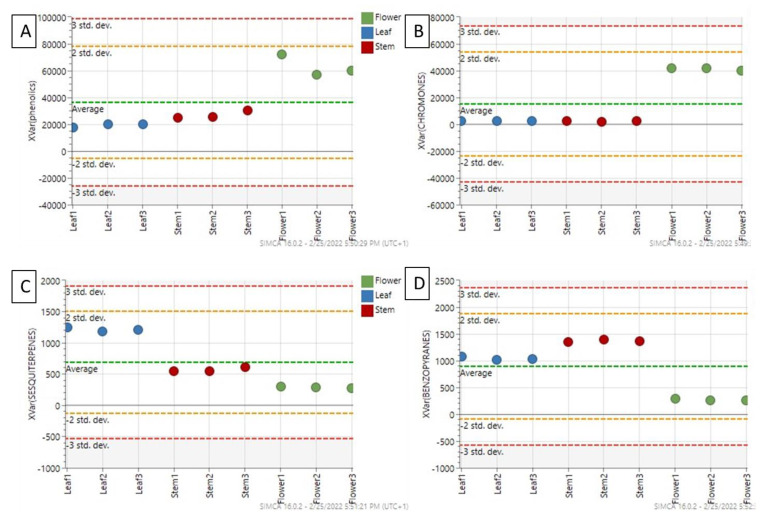
Variable trend plots of the most discriminant metabolite classes: phenolics (**A**) and chromones (**B**) are overexpressed in the flowers, while sesquiterpenes (**C**) and benzopyrans (**D**) markers had the highest influence to discriminate leaves and stems.

The untargeted profiling was carried out through UHPLC-TWINS-QTOF, to provide a comprehensive description of constituents in *R. usambarensis* stems, leaves, and flowers. The few metabolomic profiles of secondary metabolites available for this genus, namely *R. crispus, R. sanguineus, R. tunetanus*, and *R. nepalensis*, were used as references [29,30,31]. The metabolomic investigation revealed a large diversity and allowed us to annotate a total of 153 different secondary metabolites. Particularly, 58 compounds were significantly higher in flowers, 57 significantly higher in leaves, and 38 significantly higher in stems. A broad diversity of metabolites was obtained, including flavonoids, xanthones, chromones, lipids, carbohydrates, coumarins, terpenoids, and anthraquinones, as reported in the summarized table (see Table 1) (see Table S2 for all identified metabolites). With 59 different compounds, and with only 30 of them in glycosylated form, flavonoids are certainly the most represented group, as reported in Table S2 and Figure 3. Previous LC-MS/MS analysis reported a similar abundance in Rumex species other than *R. usambarensis*, confirmed by the ubiquitous nature of these secondary metabolites in the plant kingdom [32]. A slight difference in distribution throughout plant parts was noticed, as 50 flavonoids were detected in stems, 51 in leaves, and 53 in flowers, but with noticeable difference in relative intensity (see Figure 3). Not only flavonoids but all the phenolic compounds identified were significantly accumulated in flowers compared to stems and leaves, as displayed in the box plot in Figure 4A and in the histogram of Figure 3.

Flower was the organ richest in flavonoids, including seven quercetin-glycosides derivatives in negative ionization mode, as reported in a previous phytochemical profiling of Rumex genus but not yet described in *R. usambarensis* [29]. The same trend was observed for chromones (see Figure 3 and Figure 4B), whose intensities were significantly higher in flowers compared to the other organs. Xanthones were also abundant, as six different xanthones were detected in flowers, five of them with the highest intensity within this organ. These compounds are structurally related to flavonoids and commonly recovered from both plant and fungal sources [33,34]. To the best of our knowledge, this is the first time that xanthones such bellidifolin, hyperixanthone A and C, agnestin, and globusoxanthone A were detected in Rumex. Interestingly, in view of the bioassays, xanthones contribute to the antimicrobial properties of the medicinal plants producing them, thus they may be considered relevant markers for the authentication of herbal materials derived from flowers [35]. On the other hands, benzopyrans, coumarins, and sesquiterpenes were accumulated mainly in leaves and stems, as depicted in Figure 4C,D. Two anthraquinones, namely obtusifolin-glucoside and physcion-glucoside, were also identified with a significant higher intensity in leaves with respect to the other two organs. Physcion is a characteristic anthracene derivative found in Rumex genus, inasmuch as it is usual to find a high content of anthraquinones in leaves and roots with respect to other plant parts [36]. In other Rumex species, as in the case of *R. crispus* roots, the literature supports the hypothesis that multiple constituents, in particular anthraquinones, contribute to antimicrobial activity [30]. These compounds may therefore be considered as markers for such plant organs, and the concurrent absence of emodin in all samples may be of interest from a toxicological standpoint, being a toxic compound with a strict regulation in the European Union [37].

Flavonoids were also the predominant class in *R. usambarensis* leaves: 51 flavonoids were detected, including one flavo-lignan, one tannin, and two anthocyanins. To the best of our knowledge, this is the first time that sylpin (flavonol), aromadendrin-glycoside (flavonol-glycoside), comosin (isoflavone), and eriocitrin and swertiajaponin (two flavanones) were detected in *R. usambarensis*. Different organic acids were detected in leaves, including gallic acid, as previously reported for other Rumex species [29].

As expected, stem was the organ with the lower and less diverse content in secondary metabolites. Different lipid structures were identified, including long-chain fatty acids such as icosanedioic acid and octadecadynoic acid, three different hydroxycinnamic acid derivatives such ferulic acid and diferuloylgentiobiose, in agreement with previous studies on other Rumex species [29], and seven different carbohydrates and benzopyrans, whose presence discriminate stems from other organs (Figure 3 and Figure 4D).

### 2.3. Bioactivities Related to Traditional Use

Given the reported use of *R. usambarensis* aerial parts in Rwanda as a remedy for illnesses related to topical or gastrointestinal antimicrobial exposure and ROS production, specific assays were performed with leaves, stems, and flowers. All biological activities were compared to those achieved by green tea to have a reference with a polyphenol-rich plant reputed for its antioxidant and antimicrobial properties [28].

#### 2.3.1. Antioxidant Activity

In light of the differences among the wide number of test systems available, the results of a single assay can give only a reductive suggestion of antioxidant properties. Moreover, the chemical complexity of plant extracts, often a mixture of dozens of compounds with different functional groups, polarity, and chemical behavior, could lead to incongruent results according to the test employed. Among the plethora of methods that can be used for the evaluation of the antioxidant activity, very few of them (TEAC, DPPH, PCL) are useful for determining the activity of both hydrophilic and lipophilic species, thus ensuring a better comparison of the results and covering a wider range of possible applications. *Rumex usambarensis* leaves, stems, and flowers were therefore screened for their capability to scavenge the DPPH radical, followed by the determination of the total antioxidant capacity through the ABTS assay expressed as TEAC (Trolox Equivalent Antioxidant Capacity) and FRAP assay. In order to also have an estimation of the total phenolic content, Folin–Ciocalteu assay was performed.

The results reported in Table 2 show that Rumex flowers displayed a significantly higher (*p* < 0.05) antioxidant activity in both assays. This evidence agrees with the one recently provided for *R. tunetanus*, in which DPPH and FRAP values from flowers were found to be higher [29]. Overall, literature data seem to suggest that a phytocomplex richer in phenolic content may contribute to a reduction of the inflammatory status induced by many skin diseases. Such contribution has been described for extracts rich in flavonoids and low-molecular weight antioxidants, suggesting a potential anti-inflammatory topical activity promoted by their capability to locally mitigate the effects of oxidative stress. The study of Costa et al. highlights that the polyphenols of Cymbopogon citratus leaves have a potential anti-inflammatory activity, suggesting their promising application in the treatment of skin pathologies. Many other studies also suggest that polyphenols and flavonoids can protect skin from the adverse effect of UV irradiation and may be useful for skin diseases associated with solar UV-radiation-induced inflammation, oxidative stress, and DNA damage [38,39,40].

Aiming to inspect the contributions of each different class of metabolites to the biological activities we measured, Spearman’s correlation coefficients were investigated (Supplementary Table S3). Significant correlations (*p* < 0.01) were recorded between chromones and both FRAP values (0.885) and ABTS values (0.870). As mentioned in the previous paragraph, chromones were found to be markers significantly accumulated in flowers, suggesting they may be the major contributors to the higher antioxidant activity recorded for this plant part. While leaves are the most common ethnobotanical resource, likely due to their easier availability, flowers seem to provide a drug with more promising results. It must be noted, however, that *R. usambarensis* preparations were largely less effective when compared to the established commercial and experimental benchmark provided by green tea.

#### 2.3.2. Antimicrobial Activity

To further evaluate the reliability of traditional use, leaf, flower, and stem extracts were tested against the microorganisms involved in skin or intestinal infections, namely two bacterial strains (*Escherichia coli* and *Staphylococcus aureus),* and three yeasts (*Malassezia furfur*, *M. pachydermatis*, *and Candida albicans).* The only report available in the literature for leaves indicates activity against *Bacillus subtilis*, *Candida albicans*, *Mycobacterium smegmatis*, and *Staphylococcus aureus* at 1 mg/mL and no efficacy against *Salmonella gallinarum* and *Pseudomonas aeruginosa*, while water extracts obtained from stems resulted as always inactive [14].

As shown in Table 3, leaf extract was inactive against *E. coli*, whereas the flower extract showed a 51% growth inhibition at 256 µg/mL. Similar but less intense results were observed for stems with a 36% growth inhibition at 256 µg/mL (see Table 4). Regarding the leaf extract, at 16 µg/mL the 40% of *S. aureus* growth is inhibited, and the same results can be observed for flower extract, while the stem extract inhibition was 43% at 8 µg/mL. This evidence differs from a previous report in which a root extract provided no effect against *S. aureus* [14]. Overall, flower extract provided stronger effects than stem and leaf extracts, and this pattern was confirmed against specific skin pathogens such as *Candida* and *Malassezia* [26].

Flower extract confirmed a stronger antimicrobial nature, inhibiting *M. pachydermatis* and *M. furfur* growth of 92% and of 80% at 128 µg/mL, respectively. Both *Malassezia* species were the most sensitive to the exposure to *R. usambarensis* extracts as leaf extract also provided a 90% growth inhibition and an MIC of 120 ± 29.63 against *M. pachydermatis*, while offering a 73% growth inhibition at 64 µg/mL against *M. furfur* (see Table 3 and Table 4). The higher sensitivity of both *Malassezia* strains seems to support the traditional use of *R. usambarensis* and other Rumex species in the treatment of a variety of skin diseases. A good correlation (*p* < 0.01) was outlined between xanthones and *M. pachydermatis* inhibition activity, while a lack of correlation between the identified metabolites and *M. furfur* inhibition activity was observed (see Table S3).

Only the flower extract showed an activity against *Candida albicans*, with a 52% growth inhibition at the highest concentration tested, 256 µg/mL. This result is of interest, as in previous reports at 165 mg/mL a water extract obtained from the leaves of *R. usambarensis* was inactive against *Candida albicans* [17]. These data are also corroborated by the statistical analysis, because a strong correlation (*p* < 0.001) was outlined between chromones and *Candida albicans* inhibition activity, suggesting that this class of metabolites, significantly accumulated in flowers, may contribute to this biological activity.

Given the biological role of flowers and their relevance for reproduction, it is rather common to find that phytocomplexes extracted from these plant parts provide an enhanced antimicrobial defense. This emerges when flowers are compared to organs in which other strategies offer more economical solutions, such as leaves, or are less prone to bacterial aggression due to the absence of stomata, as in the case of stems.

## 3. Materials and Methods

### 3.1. Chemicals and Reagents

LC-MS grade methanol and acetonitrile were purchased from Scharlab Italia srl (Milan, Italy); bidistilled water was obtained using Milli-Q System (Millipore, Bedford, MA, USA). MS-grade ammonium acetate, acetic acid, and formic acid from Fisher Chemical (Thermo Fisher Scientific Inc., San Jose, CA, USA) was also used.

2,2-Diphenyl-1-picrylhydrazyl (DPPH), 6-hydroxy-2,5,7,8-tetramethylchroman-2-carboxylic acid (Trolox), 2,2′-azinobis(3-ethylbenzothiazoline-6-sulfonic acid) diammonium salt (ABTS), potassium persulfate, phosphate buffer, 2,4,6-tri(2-pyridyl)-s- triazine (TPTZ), iron (III) chloride hexa-hydrate, gallic acid, and Folin-Ciocalteu reagent were purchased from Sigma-Aldrich (Schnelldorf, Germany).

### 3.2. Plant Material

Leaves, flowers, and stems of *Rumex usambarensis* (Dammer) were collected in March 2020 from different plants (N = 8) grown in the botanical garden of INES-Ruhengeri, Institute of Applied Sciences, Musanze, Rwanda, where plant identification was performed by Prof. Aimable Nsanzurwimo. The different plant parts were then washed with tap water and air-dried for 3 weeks.

### 3.3. Sample Preparation

Leaves, stems (50 g), and flowers (5 g) were shredded and placed in three different hermetic flasks, independently subjected to hydroalcoholic maceration with 70% ethanol. The matrix:solvent ratio was 1:10, and the extraction process lasted for 72 h at room temperature with a magnetic stirrer at 100 rpm. After 72 h, the hydroalcoholic solutions of the three plant parts were filtered using a Buchner funnel and Whatman No.1 filter. The alcoholic part of each extract was removed using a rotary evaporator (Buchi), and the aqueous solution was frozen with liquid nitrogen and then freeze-dried. The lyophilization process lasted for 36 h, with −56 °C and 1.0 mbar (1-DL alpha Plus freeze-drier). The extracts were divided into different aliquots and reconstructed with MeOH 70% before UHPLC- TWIMS-QTOF analysis. *Camellia sinensis* var Yakubita harvested in Japan (Kirishima region) were also analyzed and used as a positive control for bioactivities [13,14,15,26,41]. Green tea leaves were extracted and freeze-dried under the same conditions used for *Rumex usambarensis.*

### 3.4. UHPLC-TWIMS-QTOF

An ACQUITY I-Class UPLC separation system coupled to a VION IMS QTOF mass spectrometer (Waters, Wilmslow, UK) equipped with an electrospray ionization (ESI) interface was employed for untargeted metabolomics. Samples were injected (1 µL) and chromatographically separated using a reversed-phase C18 BEH ACQUITY column 2.1 × 100 mm, 1.7 µm particle size (Waters, Milford, MA, USA). A gradient profile was applied using water 5 mM ammonium formiate (eluent A) and acetonitrile (eluent B), both acidified with 0.1% formic acid as mobile phases. Initial conditions were set at 5% B, after 1.5 min of isocratic step, there was a linear change to 100% B in 13.5 min. One hundred percent B was achieved in 15 min and held for 5 min to allow for column washing before returning to initial conditions. Column reconditioning was achieved over 5 min, providing a total run time of 25 min. The column was maintained at 45 °C and a flow rate of 0.35 mL/min used.

Mass spectrometry data were collected in positive and negative electrospray mode over the mass range of *m/z* 50−1000 (see Figure 1). Source settings were maintained using a capillary voltage, 1.5 kV (positive) and 2.0 kV (negative); source temperature, 120 °C; desolvation temperature, 650 °C; and desolvation gas flow, 950 L/h. The TOF analyzer was operated in sensitivity mode, and data were acquired using HDMSE, which is a data independent approach (DIA) coupled with ion mobility. The optimized ion mobility settings included: nitrogen flow rate, 90 mL/min (3.2 mbar); wave velocity, 650 m/s; and wave height, 40 V. The device within the Vion was calibrated using the Major Mix IMS calibration kit (Waters, Wilmslow, UK) to allow for CCS values to be determined in nitrogen. The calibration covered the CCS range from 130 to 306 Å2. The TOF was also calibrated prior to data acquisition and covered the mass range from 151 Da to 1013 Da. TOF and CCS calibrations were performed for both positive and negative ion mode. Data acquisition was conducted using UNIFI 1.8 (Waters, Wilmslow, UK).

#### Data Processing and Multivariate Modeling

Data processing and compound identification were conducted using Progenesis QI Informatics (Nonlinear Dynamics, Newcastle, UK). Each UHPLC-MS run was imported as an ion-intensity map, including m/z (m/z range 50–1000) and retention time, that were then aligned in the retention time direction (0–20 min). From the aligned runs, an aggregate run representing the compounds in all samples was used for peak picking. This aggregate was then compared with all runs, so that the same ions were detected in every run. Isotope and adduct deconvolution were applied to reduce the number of features detected. Unsupervised principal components analysis (PCA) with pareto scaling was performed to check the quality of the raw data. Afterward, the variables were filtered, retaining entities with coefficients of variation lower than 30% across the QCs. From the analysis of variance (ANOVA), significant features were selected, retaining those presenting, simultaneously, fold change >2 and Benjamini–Hochberg false discovery rate (FDR)-adjusted *p*-value (q-value) < 0.01. In parallel, multivariate supervised models, including partial least-squares discriminant analysis (PLSDA), were built and validated using SIMCA software (v. 16.0.2, Sartorius Stedim Data Analytics, Sweden). Cross-validation of the PLS-DA model using the one-third leaving out approach and permutation testing were applied to validate and to exclude overfitting by inspecting model parameters (goodness-of-fit R2Y and goodness of-prediction Q2Y). The variable influence in projection analysis (VIP) was further used to identify the compounds that had the highest discrimination potential (VIP value threshold > 1.2). The resulting significant features to both ANOVA *p*-values < 0.01 and VIP > 1.2 were subjected to the identification. Metabolites were identified by publicly available database searches, including Lipid Metabolites and Pathways Strategy (LIPID MAPS) [42] and Human Metabolome database (HMDB) [43], as well as by fragmentation patterns, retention times, and CCS. Based on the Metabolomics Standards Initiative [44], metabolites were annotated as level II (putatively identified compounds).

### 3.5. Evaluation of Antimicrobial/Antifungal Activity

Antimicrobial activity evaluation of *Rumex usambarensis* leaves, stems, and flowers was performed through broth microdilution assay against *Escherichia coli* ATCC 25922, *Staphylococcus aureus* ATCC 25923, *Malassezia pachydermatis* DSMZ 6172, *Malassezia furfur* ATCC 14521, and *Candida albicans* ATCC 11006. Minimal Inhibitory Concentration (MIC) evaluation was performed following the CLSI guidelines with some modifications (CLSI, 2018b). Rumex was diluted in DSMO solution at a stock concentration of 25.6 mg/mL.

#### 3.5.1. Inoculum Preparation

Reference bacterial strains were inoculated in sterile Müeller Hinton Broth (MHB) and incubated overnight at 37 °C. Yeast strains were instead inoculated in sterile RPMI broth and incubated at 37 °C for 24/48 h. The bacterial/yeast suspension was then centrifuged for 20 min at 2000 rpm and 4 °C, and then the pellet was resuspended in phosphate buffer (PB). The bacterial suspension was adjusted in PB to obtain an optical density (OD) value at 600 nm in a 1 cm light path cuvette in the range of 0.08–0.13, approximately equivalent to a 10^8^ CFU/mL suspension. The fungal suspension was adjusted to a final optical density of 0.5 McFarland standard (1–5 × 10^6^ cells/mL).

The obtained bacterial suspension was further diluted 1:100 in appropriate medium to obtain a final bacterial concentration of 10^6^ CFU/mL and inoculated within 30 min.

#### 3.5.2. MIC Assay

MIC assays were performed in 96-well microtiter plates, incubating Rumex usambarensis at concentrations ranging from 256 to 0.5 μg/mL with a final concentration of 5×10^5^ CFU/mL of bacterial suspension in a total volume of 100 μL. Growth and sterility controls were performed. For each test, three independent experiments, with three replicates each, were performed. After 24 h of incubation, the MIC value was evaluated as the arithmetic average of the lowest concentration of colistin that completely inhibited the bacterial growth as detected by the unaided eye. The standard deviation from average MIC value was also calculated. To evaluate the inhibition percentages of each tested concentration, the optical densities of each plate were read with a spectrophotometer at 620 nm. A quality control organism (*E. coli* ATCC 25922) was tested periodically to validate the accuracy of the procedure.

### 3.6. Determination of Antioxidant Activity

The antioxidant activity of the different extracts against 2,2′-azino-bis(3-ethylbenzothiazoline-6-sulphonic acid) (ABTS) and 2,2-diphenyl-1-picrylhydrazyl (DPPH) was determined using a previously described method [45,46].

Results were expressed as TEAC (Trolox Equivalent Antioxidant Capacity), which was calculated from the relation between the slope of the calibration curve and the slope of each plant extract. Ferric reducing antioxidant power (FRAP) was also calculated, and results were expressed as µM equivalent of FeSO_4_ ·7 H_2_O, calculated from the relation between the slope of the calibration curve and the slope of each plant extract [47]. In order to have an estimation of the total phenolic content in each extract, the Folin–Ciocalteau colorimetric method was perform and the results expressed as gallic acid equivalents (GAE), as previously reported [48].

### 3.7. Spectrophotometric Measurements

Absorbance measurements were taken using a Cary 60 UV-Vis Spectrophotometer (Agilent) with a wavelength range of 190–1100 nm.

### 3.8. Statistical Analysis

Statistical analyses were performed using IBM SPSS v.23.0 (SPSS Italia, Bologna, Italy) and Statistical (Tibco, Software Inc. Palo Alto, CA, USA). Data were analyzed by ANOVA followed by Tukey post-hoc test (α = 0.05).

## 4. Conclusions

To arrest the spread of pathogens, plants possess different layers of defensive responses, and some of them involve complex mixtures of secondary metabolites, including polyphenols, such as those detected in *R. usambarensis* [49]. The contribution of secondary metabolites to antimicrobial protection is related to their structure, as it is well known that simple phenolics such as pyrogallol derivatives exert a stronger and wider bioactivity [50]. Under a plant perspective, these substances act via multiple mechanisms, such as inhibition of cell envelope and nucleic acid biosynthesis, interference with biofilm formation and quorum sensing, or membrane disruption [51]. The simultaneous availability of multiple classes of metabolites may thus offer multiple ways to prevent bacterial growth, thus providing more intense result by means of a synergism in which the effect of the whole mixture may be more than the sum of its parts [6].

As for *R. usambarensis* extracts, their activity must be considered rather weak if not negligible for stems, and in most cases for leaves, while flowers seem to provide a stronger combined antioxidant and antimicrobial activity that may confirm a contribution to the ethnobotanical use reported from Rwanda. Nevertheless, overall results must be put into a context in which the recourse to a specific remedy is strongly related to the unavailability of more reliable alternatives. From the standpoint of the commercial and non-traditional use of this plant, similar extracts obtained from green tea offer stronger bioactivities.

Under a broader perspective, the data reported here suggest that the use of *R. usambarensis* flowers may provide stronger combined antioxidant and antimicrobial activity and that the authentication of products obtained from them is feasible. The presence of a more complex mixture of secondary metabolites in flowers may account for a wider range of simultaneously active mechanisms and thus for a more remarkable efficacy.

## Figures and Tables

**Table 1:** Summarized table of the significant identified metabolites with their statistical values.

Compound Name	Chemical Class	Experimental *m/z*	RT (min)	q Value	Mass Error (ppm)	Highest Mean	Fragmentation Score
PG(14:0)	Lipid	457.2542	9.7	3.1 × 10^−10^	−4.06	Flower	97
Hydroxy-phenyl--icosanone	Lipid	371.3297	15.8	7.2 × 10^−15^	−2.84	Flower	97.1
Cedeodarin	Flavonoid	317.0646	3.9	2.7 × 10^−12^	−2.85	Flower	86.2
Sylpin	Flavonoid	337.0697	4.6	7.5 × 10^−12^	4.74	Leaf	77.9
Eriocitrin	Flavonoid	635.1382	3.4	8.9 × 10^−8^	1.60	Leaf	85.2
Epicatechin-gallate	Flavonoid	713.1484	3.9	3.6 × 10^−10^	−2.33	Stem	85.4
Xanthomicrol	Flavonoid	345.0957	7.8	1.1 × 10^−10^	−3.47	Stem	82
Cinchonain Ia	Flavolignan	451.1022	4.1	7.1 × 10^−9^	−2.70	Leaf	72.7
Silandrin	Flavolignan	449.1229	7.6	5.2 × 10^−15^	−0.47	Stem	68.2
Microdiplodiasone	Chromone	259.0602	3.8	3.3 × 10^−9^	−3.59	Flower	82.1
Botrallin	Chromone	299.0552	6.5	1.3 × 10^−10^	−2.95	Leaf	84.1
Gynuraone	Chromone	177.0540	7.9	1.0 × 10^−15^	−3.18	Stem	87
Isoscopoletin	Coumarin	191.0344	3.4	1.2 × 10^−7^	−2.76	Leaf	73
Micromelin	Coumarin	269.0454	7.6	3.1 × 10^−9^	−0.37	Flower	69.3
Pratenol A	Benzopyran	241.0494	4.3	5.4 × 10^−11^	−4.81	Stem	72.2
Azanigerone E	Benzopyran	273.0734	7.5	6.2 × 10^−12^	0.19	Stem	66.1
Agnestin A	Xanthone	269.0448	6.5	8.6 × 10^−7^	−2.56	Flower	84
Bellidifolin	Xanthone	255.0290	5.3	7.7 × 10^−7^	−3.25	Flower	70.4
Nidulalin a	Xanthone	285.0749	8.2	2.9 × 10^−14^	−2.91	Leaf	84.6
Phosphoglyceroinositol	Carbohydrate	317.0641	0.7	7.3 × 10^−14^	2.61	Flower	82.8
Methyl-fusarubinlactone	Carbohydrate	333.0611	4.0	1.9 × 10^−12^	−1.44	Stem	75.4
Aloenin	Glycoside	428.1538	3.3	1.8 × 10^−10^	−3.34	Leaf	90.9
Microlenin	Sesquiterpen	477.2262	13.7	2.0 × 10^−7^	−1.96	Leaf	68.5
Hibiscoquinone A	Sesquiterpenoid	241.0849	5.1	8.6 × 10^−12^	−3.82	Leaf	68.8
Lactucin-oxalate	Sesquerpen	331.0820	6.0	1.0 × 10^−13^	2.20	Leaf	72.9
Obtusifolin glucoside	Anthraquinone	469.1110	4.3	2.3 × 10^−11^	1.07	Leaf	83.8
Physcion-glucoside	Anthraquinone	469.1117	4.2	5.2 × 10^−12^	2.73	Leaf	69.9
Prosopinine	Alkaloid	336.2521	8.2	1.1 × 10^−11^	3.79	Flower	83.9

**Table 2:** Antioxidant activities of stems, leaves, and flowers of *Rumex usambarensis* and green tea leaves hydroalcoholic extract. Data are expressed as mean ± SD of three replicates.

		ABTS-TEAC µg/mL	DPPH-TEAC µg/mL	FRAP µM	TPC (mgGAE/mL)
	Leaf	1.17 ± 0.008 ^a^	0.059 ± 0.000 ^a^	2.5 ± 0.125 ^a^	1.1 ± 0 ^a^
Rumex usambarensis	Flower	1.38 ± 0.01 ^b^	0.067 ± 0.002 ^b^	4.96 ± 0.07 ^b^	1.7 ± 0 ^b^
	Stem	0.08 ± 0.001 ^c^	0.046 ± 0.001 ^c^	0.875 ± 0 ^c^	0.63 ± 0.029 ^c^
Camellia sinensis	Leaf	11.34 ± 0.15 ^d^	0.07 ± 0.001 ^d^	8 ± 0 ^d^	2.22 ± 0.029 ^d^

Means ± SD followed by different letters are significantly different (*p* < 0.05), according to the Tukey’s post hoc test.

**Table 3:** % OD of freeze-dried extracts of *R. usambarensis* aerial parts and *C. sinensis* leaves.

		CONCENTRATION (µg/mL)									
		256	128	64	32	16	8	4	2	1	0.5
*E. coli*	RU flowers	51%	21%	−8%	−15%	−12%	−14%	−16%	−16%	−15%	−17%
	RU stems	36%	29%	18%	13%	12%	8%	3%	3%	1%	0%
	RU leaves	2%	−9%	−14%	−16%	−20%	−22%	−22%	−20%	−18%	−13%
	CS leaves	33%	34%	34%	22%	22%	15%	11%	8%	9%	7%
*S. aureus*	flowers	40%	46%	21%	−3%	−14%	−11%	−9%	−16%	2%	−5%
	stems	−152%	−69%	10%	10%	3%	43%	27%	−11%	18%	−3%
	leaves	−72%	4%	34%	40%	40%	29%	23%	8%	13%	14%
	CS leaves	84%	6%	−2%	15%	9%	28%	15%	11%	15%	0%
*C. albicans*	flowers	52%	41%	24%	1%	10%	−11%	−5%	−16%	−3%	−2%
	stems	−147%	−84%	−42%	−16%	−23%	−23%	−18%	−26%	−26%	−21%
	leaves	−66%	−34%	−26%	−36%	−24%	−21%	−48%	−33%	−37%	−37%
	CS leaves	−65%	−85%	−95%	−76%	−26%	−14%	6%	0%	0%	0%
*M. pachydermatis*	flowers	91%	92%	66%	26%	39%	32%	25%	30%	48%	28%
	stems	74%	87%	89%	82%	77%	71%	61%	56%	46%	59%
	leaves	90%	92%	86%	70%	67%	56%	57%	64%	56%	44%
	CS leaves	−27%	−79%	34%	55%	68%	75%	79%	84%	87%	81%
*M. furfur*	flowers	58%	80%	76%	57%	45%	35%	28%	11%	18%	21%
	steams	24%	56%	79%	56%	73%	68%	61%	55%	46%	38%
	leaves	29%	68%	73%	63%	43%	53%	34%	32%	23%	22%
	CS leaves	−97%	−47%	0%	0%	0%	5%	12%	10%	9%	10%

RU Rumex usambarensis, CS Camamellia sinensis. some OD% results are offset due to the inherent turbidity of the extracts.

**Table 4:** MIC of freeze-dried extracts of *R. usambarensis* aerial parts and *C.sinensis* leaves.

	*E. coli*	*S. aureus*	*C. albicans*	*M. pachydermatis*	*M. furfur*
***µg/mL* ± SD**					
*RU flowers*	256	256	>256	112 ± 29.63	256
*RU steams*	>256	>256	>256	64	256
*RU leaves*	>256	>256	>256	120 ± 22.63	>256
*CS leaves*	>256	240 ± 45.25	192 ± 68.42	≤0.5	>256

RU Rumex usambarensis, CS Camamellia sinensis.

## Data Availability

All data are contained within the article and Supplementary Materials.

## Supplementary Materials

The following supporting information can be downloaded at: https://www.mdpi.com/article/10.3390/plants12030482/s1, Table S1: ESI positive and ESI negative significant features ranked by ANOVA *p*-Value, including annotated and non-identified metabolites; Figure S1: Unsupervised principal components analysis (PCA) models built from raw positive (R^2^X 0.934; Q^2^ 0.844) (A) and negative (R^2^X 0.927; Q^2^ 0.855) (B) ionization dataset considering the two first PC (PC1 + PC2); Table S2: Significant metabolites with biochemical classes and their statistical values Table S3: Correlation matrix (Spearman) showing correlation coefficients computed for the main significant metabolite classes, antioxidant capacity, antifungal activity, and antibacterial activity.
