# Supplementary material for: Metabolite Profiling and Bioactivities of Leaves, Stems, and Flowers of Rumex usambarensis (Dammer) Dammer, a Traditional African Medicinal Plant
Source: Plants (Basel). 2023 Jan 20;12(3):482. doi: 10.3390/plants12030482 (PMC9921730; doi:10.3390/plants12030482)
Supplement: Supplementary file 1 [file plants-12-00482-s001.zip › plants-2125035-supplementary.pdf]

Supporting Information for:

Metabolite profiling and bioactivities of leaves, stems and flowers of *Rumex usambarensis* (Dammer) Dammer, a traditional African medicinal plant.

Chiara Spaggiari [1], Laura Righetti[1#], Costanza Spadini[2], Giannamaria Annunziato[1*], Aimable Nsanzurwimo[3], Clotilde Silvia Cabassi[2], Renato Bruni[1], Gabriele Costantino[1]

1 Department of Food and Drug, University of Parma, 43124 Parma, Italy
2 Department of Veterinary Science, University of Parma, 43100 Parma, Italy
3 INES-Ruhengeri, Institute of Applied Sciences, Musanze 00000, Rwanda
* Correspondence: giannamaria.annunziato@unipr.it
† Current Address: Wageningen Food Safety Research, Wageningen University & Research, P.O. Box 230, 6700 AE Wageningen, The Netherlands.
‡ Current Address: Laboratory of Organic Chemistry, Wageningen University, 6708 WE Wageningen, The Netherlands.

Table S1. ESI positive and ESI negative significant features ranked by ANOVA P-Value, including annotated and non-identified metabolites. The annotated metabolites are depicted in bold.

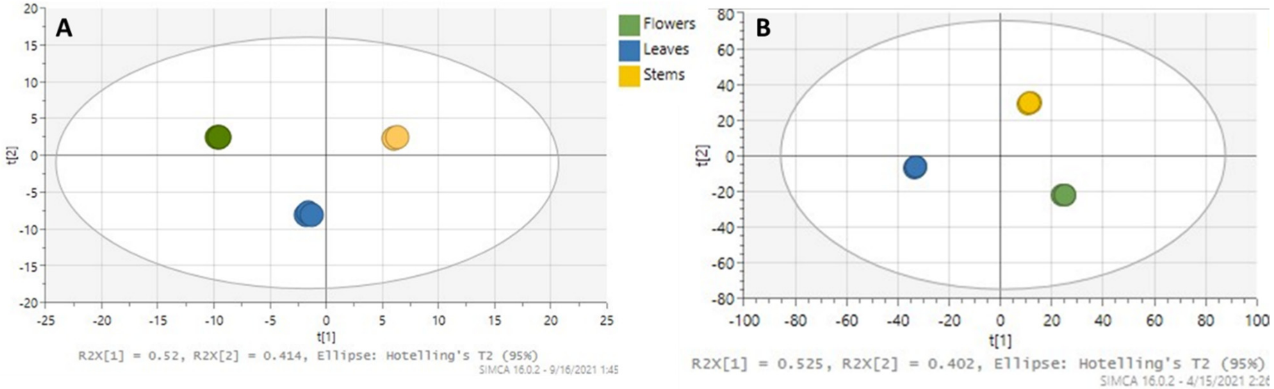

Figure S1. Unsupervised principal components analysis (PCA) models built from raw positive (R^2X 0.934; Q^2 0.844) (A) and negative (R^2X 0.927; Q^2 0.855) (B) ionization data set considering the two first PC (PC1+PC2). Green dots: flower. Yellow dots: stems. Blue dots: leaves.

Table S2: Significant metabolites with biochemical classes and their statistical values

No.	Compound name	Chemical Class	Elemental Formula	Adduct	Experimental m/z	RT (min)	q Value	Mass error (ppm)	Highest Mean	Lowest Mean	Fragmentation Score
1	PG(14:0)	Lipid	$C_{20}H_{41}O_9P$	$[M+H]^+$	457,2542	9,7	3,1E-10	-4,06	Flower	Stems	97
2	Hydroxy-phenyl--icosanone	Lipid	$C_{26}H_{44}O_2$	$[M+H^-H2O]^+$	371,3297	15,8	7,2E-15	-2,84	Flower	Leaf	97,1
3	Isanic acid	Lipid	$C_{18}H_{26}O_2$	$[M+NH_4]^+$	292,2262	6,8	4,7E-11	-3,08	Flower	Leaf	81,6
4	Sinapoyloxypalmitic acid	Lipid	$C_{27}H_{42}O_7$	$[M+FA-H]^-$	523,2911	9,2	1,3E-10	-0,34	Flower	Leaf	87,2
5	Oleoylphosphatidylethanolamine	Lipid	$C_{23}H_{46}NO_7P$	$[M-H]^-$	478,2917	10,9	1,9E-08	-4,57	Flower	Leaf	95,8
6	Linoleoyl-glycero-phosphate	Lipid	$C_{21}H_{39}O_7P$	$[M-H2O-H]^-$	415,2246	10,3	6,6E-04	-2,06	Flower	Leaf	83,6
7	PE(18:2)	Lipid	$C_{23}H_{44}NO_7P$	$[M-H]^-$	476,2773	10,6	1,2E-08	-2,09	Flower	Leaf	92,7
8	(36:6)-MGDG	Lipid	$C_{45}H_{74}O_{11}$	$[M+Na]^+$, $[M+K]^+$	813,5116	14,6	2,1E-12	-0,92	Leaf	Flower	96,9
9	MGDG(34:3)	Lipid	$C_{43}H_{76}O_{10}$	$[M+Na]^+$, $[M+K]^+$	775,5326	17,0	8,9E-14	-0,61	Leaf	Flower	99,9
10	Hexadecanoyl-glycero-phosphoserine	Lipid	$C_{22}H_{43}NO_9P^-$	$[M+FA-H]^-$	541,2673	10,5	6,8E-08	1,95	Leaf	Stem	85,6
11	Kamlonelic acid	Lipid	$C_{18}H_{30}O_3$	$[M-H2O-H]^-$	275,2008	14,6	5,0E-09	-2,69	Leaf	Flower	98,3
12	SQDG(36:6)	Lipid	$C_{45}H_{74}O_{12}S$	$[M-H]^-$	837,4819	15,7	1,6E-09	-1,05	Leaf	Stem	88,4
13	DG (22:1)	Lipid	$C_{25}H_{46}O_5$	$[M+H]^+$	427,3408	12,5	4,1E-08	-2,44	Stem	Flower	75,1
14	16:0/12-HETE PC	Lipid	$C_{44}H_{80}NO_9P$	$[M+H-H2O]^+$, $[M+Na]^+$	780,5531	14,9	1,2E-09	-0,88	Stem	Leaf	81
15	Octadecadiynoic acid	Lipid	$C_{18}H_{28}O_2$	$[M+H-H2O]^+$	259,2045	11,4	9,4E-11	-3,97	Stem	Leaf	65,1
16	Icosanedioic acid	Lipid	$C_{20}H_{38}O_4$	$[M+Na]^+$	365,2675	11,1	6,0E-11	3,60	Stem	Flower	98
17	N-(hydroxyhexadecanoyl)-sphingadienine	Lipid	$C_{34}H_{65}NO_4$	$[M-H]^-$	550,4826	15,9	8,0E-07	-2,57	Stem	Leaf	81,6
18	DG(37:5)	Lipid	$C_{40}H_{68}O_5$	$[M-H]^-$	627,4973	16,1	1,4E-05	-3,26	Stem	Flower	89,2
19	N-(hydroxyheptadecanoyl)-O-glucosyl-methylhexadecasphing-enine	Lipid	$C_{40}H_{77}NO_9$	$[M-H]^-$	714,5515	16,4	3,2E-08	-1,51	Stem	Leaf	81,7
20	Ceramide	Lipid	$C_{33}H_{65}NO_3$	$[M+FA-H]^-$	568,4938	16,6	9,3E-10	-1,62	Stem	Leaf	75,1
21	Methoxyluteolin	Flavonoid	$C_{16}H_{12}O_7$	$[M+H]^+$	317,0646	6,8	0,0E+00	-3,01	Flower	Leaf	77,4
22	Annagenin	Flavonoid	$C_{16}H_{12}O_8$	$[M+H]^+$	333,0596	6,1	0,0E+00	-2,68	Flower	Leaf	79,1
23	Cedeodarin	Flavonoid	$C_{16}H_{14}O_8$	$[M+H-H2O]^+$	317,0646	3,9	2,7E-12	-2,85	Flower	Stem	86,2
24	Naringenin	Flavonoid	$C_{15}H_{12}O_5$	$[M+K]^+$	291,0897	5,2	2,7E-08	0,34	Flower	Stem	72,3

25	Annulatin	Flavonoid	$C_{16}H_{12}O_{8}$	$[M+H]^{+}$	333,0595	4,3	3,7E-12	-2,94	Flower	Leaf	80,4
26	Pinoquercetin	Flavonoid	$C_{16}H_{12}O_{7}$	$[M+H]^{+}$	317,0649	11,8	3,5E-15	-2,00	Flower	Leaf	95,8
27	Plantagoside	Flavonoid	$C_{21}H_{22}O_{12}$	$[M+H-H2O]^{+}$	449,1071	3,2	5,7E-17	-1,52	Flower	Stem	82
28	Quercetin-O-rhamnofuranoside	Flavonoid	$C21H_{20}O_{11}$	$[M+H]^{+}$	449,1074	0,8	2,0E-12	-0,86	Flower	Stem	83,9
29	Quercetin-olate	Flavonoid	$C_{15}H_{9}O_{7}^{-}$	$[M+NH_{4}]^{+}$	319,0705	5,0	1,3E-08	4,28	Flower	Leaf	70,8
30	Scutellarein	Flavonoid	$C_{15}H_{10}O_{6}$	$[M+H]^{+}$	287,0562	3,8	8,9E-09	4,23	Flower	Stem	89,6
31	Acetylastragalin	Flavonoid	$C_{23}H_{22}O_{12}$	$[M-H]^{-}$	489,1028	6,9	7,7E-11	-2,02	Flower	Stem	75,1
32	Dihydromyrcetin	Flavonoid	$C_{15}H_{11}O_{8}^{-}$	$[M-H2O-H]^{-}$	300,0265	4,6	7,9E-07	-4,88	Flower	Leaf	78,9
33	Kaempferol -rhamnosyl-rutinoside	Flavonoid	$C_{33}H_{40}O_{19}$	$[M+FA-H]^{-}$	785,2130	3,4	4,6E-10	-2,44	Flower	Leaf	75,8
34	Ampeloptin	Flavonoid	$C_{15}H_{12}O_{8}$	$[M-H2O-H]^{-}$	301,0339	4,5	7,4E-06	-4,56	Flower	Leaf	90,8
35	Datiscetin	Flavonoid	$C_{15}H_{10}O_{6}$	$[M-H]^{-}$	285,0397	6,2	7,7E-10	-2,63	Flower	Stem	68,7
36	Dihydrogossypetin	Flavonoid	$C_{15}H_{12}O_{8}$	$[M-H2O-H]^{-}$	301,0339	5,7	9,0E-05	-4,57	Flower	Leaf	90,7
37	Dihydrotricetin	Flavonoid	$C_{15}H_{12}O_{7}$	$[M-H2O-H]^{-}$	285,0395	5,6	1,8E-08	-3,18	Flower	Stem	71,7
38	Hypolaetin	Flavonoid	$C_{15}H_{10}O_{7}$	$[M-H]^{-}$	301,0341	5,7	1,2E-06	-4,28	Flower	Leaf	82,9
39	Isoquercetin/Quercetin-glucoside	Flavonoid	$C_{21}H_{20}O_{12}$	$[M-H]^{-}$	463,0874	5,3	3,2E-08	-1,67	Flower	Leaf	88,9
40	Kaempferol-neohesperidoside	Flavonoid	$C_{27}H_{30}O_{15}$	$[M-H]^{-}$	593,1500	4,7	7,1E-07	-2,05	Flower	Stem	76,4
41	Luteolin	Flavonoid	$C_{15}H_{10}O_{6}$	$[M+FA-H]^{-}$	331,0448	5,8	7,5E-10	-3,97	Flower	Leaf	75,6
42	Myricitin	Flavonoid	$C_{21}H_{20}O_{12}$	$[M-H]^{-}$	463,0867	3,4	4,9E-09	-3,17	Flower	Leaf	81,2
43	Quercetin	Flavonoid	$C_{21}H_{20}O_{11}$	$[M-H]^{-}$	447,0923	3,7	1,3E-09	-2,13	Flower	Stem	88,6
44	Quercetin-glicoside	Flavonoid	$C_{27}H_{30}O_{16}$	$[M-H]^{-}$	609,1451	4,5	3,0E-06	-1,69	Flower	Leaf	96,3
45	Rutin	Flavonoid	$C_{27}H_{30}O_{16}$	$[M-H2O-H]^{-}$	591,1325	4,7	2,8E-05	-4,95	Flower	Stem	77,3
46	Tiliroside	Flavonoid	$C_{30}H_{26}O_{13}$	$[M-H]^{-}$	593,1295	6,1	3,1E-09	-0,90	Flower	Stem	78,2
47	Epigallocatechin	Flavonoid	$C_{30}H_{26}O_{13}$	$[M+H-H2O]^{+}$	577,1312	0,7	4,9E-08	-4,81	Leaf	Flower	92,1
48	Sylpin	Flavonoid	$C_{17}H_{14}O_{6}$	$[M+Na]^{+}$	337,0697	4,6	7,5E-12	4,74	Leaf	Flower	77,9
49	Aromadendrin-galactoside	Flavonoid	$C_{21}H_{22}O_{11}$	$[M+H-H2O]^{+}$	433,1116	5,2	2,0E-10	-2,92	Leaf	Flower	97,1
50	Kaempferol-O-coumaroyl-rhamnopyranoside	Flavonoid	$C_{30}H_{26}O_{12}$	$[M+H]^{+}$	579,1485	3,6	1,5E-14	-2,12	Leaf	Stem	68,6
51	Acetylglycetin	Flavonoid	$C_{24}H_{24}O_{11}$	$[M+Na]^{+}$	511,1223	4,2	5,1E-13	2,39	Leaf	Flower	65,3
52	Comosin	Flavonoid	$C_{19}H_{18}O8$	$[M+Na]^{+}$	397,0900	4,8	6,5E-12	1,74	Leaf	Flower	78,7
53	Eriocitrin	Flavonoid	$C_{27}H_{32}O_{15}$	$[M+K]^{+}$	635,1382	3,4	8,9E-08	1,60	Leaf	Flower	85,2
54	Isohemiphloin	Flavonoid	$C_{21}H_{22}O_{10}$	$[M+Na]^{+}$	457,1117	4,3	2,1E-10	2,74	Leaf	Flower	69,3
55	Kaempferol	Flavonoid	$C_{15}H_{10}O_{6}$	$[M+H]^{+}$	287,0542	6,4	1,1E-15	-2,68	Leaf	Stem	69

56	Kaempferol-rhamnoglycoside	Flavonoid	$C_{27}H_{30}O_{15}$	$[M+H]^+$	595,1485	0,8	3,1E-12	-2,06	Leaf	Stem	67,1
57	Luteolin-rhamnoside	Flavonoid	$C_{21}H_{20}O_{10}$	$[M+Na]^+$	455,0937	5,4	1,2E-12	-2,62	Leaf	Stem	76,6
58	Methyl hesperidin	Flavonoid	$C_{29}H_{36}O_{15}$	$[M+K]^+$	663,1714	3,4	1,3E-12	4,50	Leaf	Flower	71
59	Swertiajaponin	Flavonoid	$C_{22}H_{22}O_{11}$	$[M+H-H2O]^+$, $[M+H]^+$	463,1227	4,8	9,4E-11	-1,73	Leaf	Flower	77,9
60	Taxifolin acetate	Flavonoid	$C_{17}H_{14}O_8$	$[M-H2O-H]^-$	327,0503	5,0	5,3E-07	-1,98	Leaf	Flower	84,3
61	Acetyldaidzin	Flavonoid	$C_{23}H_{22}O_{10}$	$[M+FA-H]^-$	503,1181	4,2	8,6E-08	-3,16	Leaf	Flower	77
62	Methylderhamnosylmaysin	Flavonoid	$C_{22}H_{20}O_{10}$	$[M+H-H2O]^+$	427,1007	4,8	1,5E-14	-3,69	Leaf	Flower	80,1
63	Kaempferol	Flavonoid	$C_{15}H_{10}O_6$	$[M-H2O-H]^-$, $[M-H]^-$	285,0392	6,4	9,9E-10	-4,84	Leaf	Stems	73,7
64	Epicatechin-gallate	Flavonoid	$C_{37}H_{30}O_{16}$	$[M+H-H2O]^+$	713,1484	3,9	3,6E-10	-2,33	Stem	Flower	85,4
65	Dihydrodaidzein-glucuronide	Flavonoid	$C_{21}H_{20}O_{10}$	$[M+H]^+$	433,1120	4,6	3,4E-13	-2,09	Stem	Flower	65,5
66	Oxo-phenyl-chromen-glucopyranoside	Flavonoid	$C_{21}H_{20}O_8$	$[M+H-H2O]^+$	383,1115	7,7	5,0E-15	-2,58	Stem	Leaf	84,2
67	Hydroxyflavanone-O-glucoside	Flavonoid	$C_{21}H_{22}O_8$	$[M+H-H2O]^+$	385,1270	6,9	3,4E-13	-2,82	Stem	Flower	95,4
68	Cacticin	Flavonoid	$C_{22}H_{22}O_{12}$	$[M+H]^+$	479,1172	5,1	5,5E-10	-2,42	Stem	Flower	74,5
69	Ferreirin	Flavonoid	$C_{16}H_{14}O_6$	$[M+H]^+$	303,0855	6,9	1,6E-11	-2,80	Stem	Flower	74,8
70	Myrciacitrin II	Flavonoid	$C_{24}H_{28}O_{11}$	$[M+Na]^+$	515,1540	6,2	1,9E-12	3,36	Stem	Flower	79,5
71	Xanthomicrol	Flavonoid	$C_{18}H_{16}O_7$	$[M+H]^+$	345,0957	7,8	1,1E-10	-3,47	Stem	Leaf	82
72	Apigenin-glucoside	Flavonoid	$C_{23}H_{22}O_{11}$	$[M-H2O-H]^-$	455,0976	7,7	1,7E-08	-1,53	Stem	Flower	78,1
73	Methoxyaromadendrin-acetate	Flavonoid	$C_{18}H_{16}O_8$	$[M-H2O-H]^-$	341,0653	4,6	4,8E-10	-3,81	Stem	Leaf	75,1
74	Kaempferol-rhamnoside	Flavonoid	$C_{21}H_{20}O_{10}$	$[M-H]^-$	432,1850	5,5	1,0E-10	-4,08	Stem	Flower	73,2
75	Cinchonain Ia	Flavolignan	$C_{24}H_{20}O_9$	$[M-H]^-$	451,1022	4,1	7,1E-09	-2,70	Leaf	Flower	72,7
76	Silandrin	Flavolignan	$C_{25}H_{22}O_9$	$[M+H-H2O]^+$	449,1229	7,6	5,2E-15	-0,47	Stem	Leaf	68,2
77	Procyanidin -O-gallate	Tannin	$C_{37}H_{30}O_{16}$	$[M+H]^+$	731,1600	0,8	1,0E-11	-0,92	Leaf	Stem	90,9
78	Pelargonidin	Antocyanin	$C_{15}H_{12}O_5$	$[M-H2O-H]^-$	253,0493	6,5	8,0E-11	-4,79	Stem	Leaf	70,7
79	Pelargonidin di glucoside	Antocyanin	$C_{27}H_{30}O_{15}$	$[M+Na]^+$	617,1462	4,8	7,6E-08	-2,47	Leaf	Flower	69,5
80	Dihydroxy-dimethoxy-methyl-dihydro-benzoisochromenetrione	Chromone	$C_{16}H_{14}O_8$	$[M+H-H2O]^+$	317,0649	4,5	1,3E-14	-2,06	Flower	Leaf	88,8
81	Aloesin	Chromone	$C_{19}H_{22}O_9$	$[M+K]^+$	433,0897	5,2	2,7E-08	0,34	Flower	Stem	72,3
82	Microdiplodiasone	Chromone	$C_{14}H_{14}O_6$	$[M-H2O-H]^-$	259,0602	3,8	3,3E-09	-3,59	Flower	Stem	82,1

83	Botrallin	Chromone	$C_{16}H_{14}O_7$	$[M-H2O-H]^-$	299,0552	6,5	1,3E-10	-2,95	Leaf	Flower	84,1
84	Cyathusal B	Chromone	$C_{17}H_{14}O_8$	$[M-H2O-H]^-$	327,0497	8,5	1,3E-10	-3,86	Leaf	Flower	70,3
85	Gynuraone	Chromone	$C_{10}H_{10}O_4$	$[M+H-H2O]^+$	177,0540	7,9	1,0E-15	-3,18	Stem	Leaf	87
86	Isoscopoletin	Coumarin	$C_{10}H_8O_4$	$[M-H]^-$	191,0344	3,4	1,2E-07	-2,76	Leaf	Stem	73
87	Neobyakangelicol	Coumarin	$C_{17}H_{16}O_6$	$[M-H2O-H]^-$	297,0757	6,4	2,9E-08	-3,75	Stem	Flower	67,8
88	MUG	Coumarin	$C_{16}H_{18}O_8$	$[M+Na]^+$	361,0903	9,7	8,2E-17	2,65	Leaf	Flower	96,1
89	Micromelin	Coumarin	$C_{15}H_{12}O_6$	$[M-H2O-H]^-$	269,0454	7,6	3,1E-09	-0,37	Flower	Stem	69,3
90	Hispidin	Phenol derivative	$C_{13}H_{10}O_5$	$[M-H2O-H]^-$	227,0342	5,4	6,8E-07	-3,37	Leaf	Stem	92,3
91	Coriandrone E	Benzopyran	$C_{13}H_{12}O_5$	$[M-H2O-H]^-$	229,0494	3,8	1,4E-09	-4,89	Flower	Leaf	65,2
92	Swerilactone N	Benzopyran	$C_{13}H_{14}O_4$	$[M+NH_4]^+$	252,1219	3,7	5,7E-12	-4,91	Flower	Stem	87
93	Grandmarin	Benzopyran	$C_{15}H_{16}O_6$	$[M+Na]^+$	315,0826	9,4	1,6E-11	-4,48	Leaf	Flower	78,4
94	Pratenol B	Benzopyran	$C_{15}H_{12}O_7$	$[M-H2O-H]^-$	285,0399	6,8	9,1E-05	-1,87	Leaf	Stem	93,5
95	Pratenol A	Benzopyran	$C_{14}H_{12}O_5$	$[M-H2O-H]^-$	241,0494	4,3	5,4E-11	-4,81	Stem	Leaf	72,2
96	Azanigerone E	Benzopyran	$C_{13}H_{14}O_5$	$[M+Na]^+$	273,0734	7,5	6,2E-12	0,19	Stem	Leaf	66,1
97	fulvoplumierin	Pyran	$C_{14}H_{12}O_4$	$[M-H2O-H]^-$	225,0547	4,5	7,1E-10	-4,17	Stem	Leaf	75,8
98	Globosuxanthone A	Xanthone	$C_{15}H_{12}O_7$	$[M+H-H2O]^+$	287,0540	4,3	6,8E-11	-3,41	Flower	Leaf	85,6
99	Hyperixanthone A	Xanthone	$C_{28}H_{32}O_6$	$[M+H]^+$	447,2150	14,0	9,2E-12	-3,32	Flower	Stem	72,9
100	Hyperxanthone C	Xanthone	$C_{18}H_{16}O_7$	$[M+H]^+$	345,0954	5,4	1,7E-10	-4,33	Flower	Leaf	65,2
101	Agnestin A	Xanthone	$C_{15}H_{12}O_6$	$[M-H2O-H]^-$	269,0448	6,5	8,6E-07	-2,56	Flower	Leaf	84
102	Bellidifolin	Xanthone	$C_{14}H_{10}O_6$	$[M-H2O-H]^-$	255,0290	5,3	7,7E-07	-3,25	Flower	Stem	70,4
103	Nidulalin a	Xanthone	$C_{16}H_{14}O_6$	$[M+H-H2O]^+$	285,0749	8,2	2,9E-14	-2,91	Leaf	Flower	84,6
104	Carthamone	Hydroxycinnamic acid derivative	$C_{21}H_{20}O_{11}$	$[M-H]^-$	447,0916	6,2	1,5E-09	-3,66	Flower	Stem	84,6
105	Ferulic acid	Hydroxycinnamic acid derivative	$C_{10}H_{10}O_4$	$[M+H-H2O]^+$	177,0540	6,9	1,2E-10	-2,99	Stem	Flower	70,3
106	Diferuloylgentiobiose	Hydroxycinnamic acid derivative	$C_{32}H_{38}O_{17}$	$[M+H-H2O]^+$, $[M+Na]^+$	717,1991	5,6	1,5E-13	-1,43	Stem	Leaf	91,2
107	Succinyl-Hydroxycyclohexa-diene-carboxylic acid	Organic acid	$C_{11}H_{12}O_6$	$[M-H2O-H]^-$	221,0444	3,9	3,6E-13	-4,91	Flower	Stem	75,3
108	Tetracetic acid lactone	Organic acid	$C_8H_8O_4$	$[M-H]^-$	167,0342	3,2	7,5E-10	-4,73	Flower	Stem	82

109	Amburoside A	Organic acid	$C_{20}H_{22}O_{10}$	$[M+Na]^+$	445,1097	4,9	3,7E-08	-1,91	Leaf	Flower	69,3
110	Hydroxyketononatrienedionate	Organic acid	$C_9H_8O_6$	$[M-H2O-H]^-$	193,0133	3,4	1,3E-10	-4,59	Stem	Flower	82,1
111	Gallic acid	Organic acid	$C_7H_6O_5$	$[M-H]^-$	169,0135	1,2	2,9E-10	-4,54	Leaf	Stem	95,7
112	(Cyclopentylcarbamoyl)amino]-(hydroxymethyl)-tetrahydro-pyranobenzofuran(methoxybenzyl)acetamide	Organic compound	$C_{28}H_{35}N_3O_6$	$[M+H-H2O]^+$	492,2484	14,0	5,6E-13	-1,82	Leaf	Stem	79,6
113	Acetyl-(acetyl-trihydroxy-methylphenyl)-dihydroxy-methyl-cyclohexadien-one	Organic compound	$C_{18}H_{18}O_8$	$[M+H-H2O]^+$	345,0958	9,0	1,0E-12	-3,02	Leaf	Flower	77,3
114	Phosphoglyceroinositol	Carbohydrate	$C_9H_{19}O_{11}P$	$[M+H-H2O]^+$	317,0641	0,7	7,3E-14	2,61	Flower	Leaf	82,8
115	Tetrasaccharide	Carbohydrate	$C_{24}H_{42}O_{21}$	$[M-H]^-$	665,2136	0,9	1,0E-11	-1,48	Flower	Stem	93,7
116	Methylacetyl-galactoside	Carbohydrate	$C_{11}H_{18}O_8$	$[M+K]^+$	317,0646	3,6	8,5E-12	4,68	Flower	Leaf	89,3
117	Deoxy-manno-oct-ulopyranosonic acid-phosphate	Carbohydrate	$C_8H_{15}O_{11}P$	$[M-H2O-H]^-$	299,0186	7,5	2,4E-12	3,92	Leaf	Flower	67,4
118	D-galactopyranose	Carbohydrate	$C_{36}H_{61}N_3O_{25}$	$[M+H]^+$	936,3671	7,6	3,2E-17	0,40	Stem	Flower	70,1
119	Amino-tetrasaccaride	Carbohydrate	$C_{31}H_{52}N_2O_{24}$	$[M+Na]^+$	859,2766	7,0	9,8E-16	-4,30	Stem	Leaf	76,4
120	Methyl-fusarubinlactone	Carbohydrate	$C_{16}H_{14}O_8$	$[M-H]^-$	333,0611	4,0	1,9E-12	-1,44	Stem	Flower	75,4
121	Aloenin	Glycoside	$C_{19}H_{22}O_{10}$	$[M+NH_4]^+$	428,1538	3,3	1,8E-10	-3,34	Leaf	Flower	90,9
122	GalP-mannopyranoside	Posphono mannopyranoside	$C_{12}H_{23}O_{14}P$	$[M+Na]^+$	445,0738	0,7	1,2E-10	4,82	Leaf	Flower	76,1
123	Microlenin	Sesquiterpen	$C_{29}H_{34}O_7$	$[M+H-H2O]^+$	477,2262	13,7	2,0E-07	-1,96	Leaf	Stem	68,5
124	Hibiscoquinone A	Sesquiterpenoid	$C_{15}H_{14}O_4$	$[M+H-H2O]^+$	241,0849	5,1	8,6E-12	-3,82	Leaf	Stem	68,8
125	Lactucin-oxalate	Sesquerpen	$C_{17}H_{16}O_8$	$[M+H-H2O]^+$	331,0820	6,0	1,0E-13	2,20	Leaf	Stem	72,9
126	Obtusifolin glucoside	Anthraquinone	$C_{22}H_{22}O_{10}$	$[M+Na]^+$	469,1110	4,3	2,3E-11	1,07	Leaf	Flower	83,8
127	Physcion -glucoside	Anthraquinone	$C_{22}H_{22}O_{10}$	$[M+Na]^+$	469,1117	4,2	5,2E-12	2,73	Leaf	Flower	69,9
128	Prosopinine	Alkaloid	$C_{18}H_{35}NO_3$	$[M+Na]^+$	336,2521	8,2	1,1E-11	3,79	Flower	Leaf	83,9
129	Uvaricin	Acetogenin	$C_{39}H_{68}O_7$	$[M+Na]^+$	671,4847	14,7	1,2E-10	-1,56	Flower	Leaf	73,9

130	N-methoxy-trimethyl-oxo-(pyridinylmethyl)-oxa-diazabicyclohexadeca-trien-methylbenzenesulfonamide	Benzensulfonamide	$C_{30}H_{38}N_4O_5S$	$[M+Na]^+$	589,2441	0,6	2,3E-14	-2,41	Flower	Leaf	71,2
131	Epi-valiolone-phosphate	Organophosphate oxoanion	$C_7H_{11}O_9P^-{}_2$	$[M+FA-H]^-$	315,0131	4,6	1,3E-09	-0,97	Flower	Leaf	76,7
132	Agrocinopine B	Agrocinopines	$C_{11}H_{21}O_{13}P$	$[M-H2O-H]^-$	373,0541	4,5	1,5E-08	-0,14	Flower	Leaf	89,5
133	Histidinate	Amino acid anion	$C_6H_8N_3O_2^-$	$[M-H2O-H]^-$	135,0440	2,4	1,3E-08	-2,03	Flower	Stem	79,6
134	Validone-phosphate	Cyclitolo phosphate	$C_7H_{13}O_8P$	$[M+FA-H]^-$	301,0324	4,8	5,9E-08	-2,24	Flower	Leaf	91,9
135	Purpurquinone C	Azaphilone	$C_{21}H_{20}O_8$	$[M+NH_4]^+$	418,1485	4,4	4,0E-13	-2,74	Leaf	Stem	65,3
136	SEK4b	Poliketide	$C_{16}H_{14}O_7$	$[M+H-H2O]^+$	301,0697	7,9	2,5E-12	-3,00	Leaf	Flower	88,5
137	Gaudimycin B	Benzoantrachene	$C_{19}H_{16}O_7$	$[M+Na]^+$	379,0802	4,2	3,4E-14	3,95	Leaf	Flower	71,6
138	Methyl-gingerol	Hydroxy ketone	$C_{22}H_{36}O_4$	$[M+H]^+$	365,2677	12,9	2,4E-10	-2,54	Leaf	Flower	95,7
139	Red chlorophyll catabolite	Macrocycle	$C_{35}H_{38}N_4O_7$	$[M+H]^+$, $[M+Na]^+$	627,2802	9,5	1,2E-13	-1,85	Leaf	Stem	90,2
140	Cerbertin	Cardiac glycoside	$C_{32}H_{44}O_{11}$	$[M+Na]^+$	627,2801	9,9	1,9E-14	4,13	Leaf	Stem	96,3
141	Chisomicine A	Limonoid	$C_{32}H_{38}O_8$	$[M+H-H2O]^+$	533,2537	13,9	4,9E-12	0,66	Leaf	Stem	75,4
142	Scilliroside	Steroid glycoside	$C_{32}H_{44}O_{12}$	$[M+Na]^+$	643,2745	8,9	3,5E-10	3,17	Leaf	Stem	87,7
143	Palmitoylglycerophosphoinositol	Glycerophosphoinositols	$C_{25}H_{49}O_{12}P$	$[M-H]^-$	571,2879	10,5	2,5E-06	-1,78	Leaf	Flower	92,2
144	Caproyl-sn-glycero-phosphate	Glycerophosphate	$C_9H_{19}O_7P$	$[M+FA-H]^-$	315,0840	4,7	2,3E-05	-4,06	Leaf	Flower	76
145	Auramycinone	Anthracycline	$C_{21}H_{18}O_8$	$[M+FA-H]^-$	443,0970	4,2	4,1E-08	-3,32	Leaf	Flower	72
146	Funalenone	Phenalene	$C_{15}H_{12}O_6$	$[M-H2O-H]^-$	269,0448	8,8	2,5E-10	-2,70	Leaf	Flower	72,9
147	Acetylshikonin	Naphtoquinone	$C_{18}H_{18}O_6$	$[M+H-H2O]^+$	313,1064	11,0	3,5E-12	-2,14	Stem	Flower	91,3
148	Filicinic Acid	Enone	$C_8H_{10}O_3$	$[M+Na]^+$	177,0522	8,0	1,7E-12	0,19	Stem	Leaf	78,4
149	Decadienoyl-CoA	Enoyl coas	$C_{31}H_{50}N_7O_{17}P_3S$	$[M+NH_4]^+$	935,2546	7,2	1,3E-14	1,16	Stem	Flower	72,1

150	Julichrome Q6	Anthracene	$C_{19}H_{20}O_6$	$[M+H-H2O]^+$	327,1218	11,5	8,8E-15	-2,45	Stem	Flower	93,2
151	Misoprostol	Anologue prostaglandin E1	$C_{22}H_{38}O_5$	$[M+H-H2O]^+$	365,2677	11,3	5,7E-11	-2,37	Stem	Flower	92,2
152	Fulvoplumierin	Pyran	$C_{14}H_{12}O_4$	$[M-H2O-H]^-$	225,0547	4,5	7,1E-10	-4,17	Stem	Flower	75,8
153	Methylstyrylpyron	Styrene derivative	$C_{14}H_{12}O4$	$[M-H2O-H]^-$	225,0548	7,7	2,0E-05	-3,74	Stem	Flower	73

Table S3. Correlation matrix (Sperman) showing correlation coefficients computed for the main significant metabolite classes, antioxidant capacity, antifungal activity and antibacterial activity.

Variable	Phenolics	Chromones	Sesquiterpenes	Coumarin	Benzopyranes	Xanthones	Antifungal activity: Candida Albicans	Antifungal activity: Malassezia Furfur	Antifungal activity: Malassezia Pachydermatis	Antibacterial activity: E. Coli	Antibacterial activity: S. Aureus	Antioxidant activity: DPPH TEAC	Antioxidant activity: ABTS TEAC	Antioxidant activity: FRAP ASSAY	Antioxidant activity: TPC
Phenolics	1.000	0.617	-0.883 **	-0.367	-0.433	-0.417	-0.600	0.467	-0.150	-0.783 *	0.183	0.444	0.460	0.468	0.442
Chromones	0.617	1.000	-0.583	-0.700 *	-0.917 ***	0.217	-0.917 ***	-0.283	-0.433	-0.467	-0.483	0.795 *	0.870 **	0.885 **	0.797
Sesquiterpenes	-0.883 **	-0.583	1.000	0.450	0.517	0.483	0.450	-0.483	-0.050	0.833 **	-0.350	-0.527	-0.460	-0.519	-0.494
Coumarin	-0.367	-0.700 *	0.450	1.000	0.883 **	-0.433	0.767 *	0.333	0.333	0.550	0.200	-0.929 ***	-0.895 ***	-0.911 ***	-0.962 ***
Benzopyranes	-0.433	-0.917 ***	0.517	0.883 **	1.000	-0.400	0.850 **	0.417	0.350	0.467	0.383	-0.929 ***	-0.954 **	-0.979 ***	-0.936 ***
Xanthones	-0.417	0.217	0.483	-0.433	-0.400	1.000	-0.400	-0.867 **	-0.100	0.433	-0.633	0.452	0.427	0.443	0.442
Antifungal activity (Candida Albicans)	-0.600	-0.917 ***	0.450	0.767 *	0.850 **	-0.400	1.000	0.333	0.450	0.417	0.533	-0.828 **	-0.862 **	-0.877 **	-0.840 **
Antifungal activity (Malassezia Furfur)	0.467	-0.283	-0.483	0.333	0.417	-0.867 **	0.333	1.000	0.283	-0.550	0.800 **	-0.377	-0.460	-0.434	-0.321
Aantifungal activity (Malassezia Pachydermatis)	-0.150	-0.433	-0.050	0.333	0.350	-0.100	0.450	0.283	1.000	0.233	0.467	-0.201	-0.469	-0.323	-0.329
Antibacterial activity (Coli)	-0.783 *	-0.467	0.833 **	0.550	0.467	0.433	0.417	-0.550	0.233	1.000	-0.467	-0.460	-0.460	-0.451	-0.563
Antibacterial activity (Aureus)	0.183	-0.483	-0.350	0.200	0.383	-0.633	0.533	0.800 **	0.467	-0.467	1.000	-0.276	-0.393	-0.375	-0.234
DPPH TEAC	0.444	0.795 *	-0.527	-0.929 ***	-0.929 ***	0.452	-0.828 **	-0.377	-0.201	-0.460	-0.276	1.000	0.899 ***	0.975 ***	0.966 ***

Spearman Rank Order Correlations

ABTS TEAC	0.460	0.870	**	-0.460	-0.895	**	-0.954	***	0.427	-0.862	**	-0.460	-0.469	-0.460	-0.393	0.899	***	1.000		0.966	***	0.914	***
FRAP ASSAY	0.468	0.885	**	-0.519	-0.911	***	-0.979	***	0.443	-0.877	**	-0.434	-0.323	-0.451	-0.375	0.975	***	0.966	***	1.000		0.956	***
TPC	0.442	0.797		-0.494	-0.962	***	-0.936	***	0.442	-0.840	**	-0.321	-0.329	-0.563	-0.234	0.966	***	0.914	***	0.956	***	1.000	

* Significant correlation p > 0.05

** Significant correlation p > 0.01

***Significant correlation p > 0.001
